# Chicago Veterinary College.—A New Veterinary Degree

**Published:** 1893-12

**Authors:** 


### CHICAGO VETERINARY COLLEGE.

### A NEW VETERINARY DEGREE.

The degree given by the Chicago Veterinary College has been changed from *Doctor of Veterinary Science* (D. V. S.) to *Doctor of Comparative Medicine* (M. D. C.)

This change was made at the unanimous request of the classes of '92 and '93 and eighty-five per cent. of the graduates. The main reasons given for the change are that M. D. C. is more appropriate, comprehensive, elevating, and less liable to be assumed by empirical practitioners than V. S., or any other combination of initials relating to the title.

Former graduates can avail themselves of the change by returning to the Secretary their old diplomas accompanied by five dollars, the cost of the new ones. Excepting the change in degree, the new diplomas will be as far as practicable fac-similes of the old.

The first diplomas bearing the new title were conferred on March 24th, 1893.

